# Innovation workshop using design thinking framework and involving stakeholders to co-create ideas for management of asthma

**DOI:** 10.1038/s41533-023-00357-4

**Published:** 2023-11-04

**Authors:** Mabel Qi He Leow, Aminath Shiwaza Moosa, Hani Salim, Adina Abdullah, Yew Kong Lee, Chirk Jenn Ng, Ngiap Chuan Tan

**Affiliations:** 1 SingHealth Polyclinics, Singapore, Singapore; 2 Universiti Putra Malaysia, Serdang, Malaysia; 3 Universiti Malaya, Kuala Lumpur, Malaysia; 4 Duke-NUS Medical School, Singapore, Singapore

**Keywords:** Health services, Respiratory tract diseases

## Abstract

This paper described the use of photovoice within design thinking to empathise with patients’ challenges and co-create ideas on asthma management in Singapore. A one-day workshop was organised and conducted in Singapore by SingHealth Polyclinics to discuss the challenges and enablers of good asthma care and ideate innovations to address the issues discussed. The workshop was conceptualised based on the Stanford’s d: school Design Thinking Process: 1. empathise, 2. define, 3. ideate, 4. prototype, and 5. test, focussing on the first three stages. Empathise stage was executed by having two patients share their challenges and enablers of good asthma care using photovoice. Define and ideate stage were accomplished through the multidisciplinary team discussion, with the patient going to every group to allow them to seek clarifications and opinions on ideas. The study findings were summarised based on the Empathise, Define and Ideate stages. Thirty-seven healthcare providers attended—9 doctors, 14 nurses, 4 pharmacists, 3 clinical service, 3 medical students and 4 research staff. Participants’ feedback was collected via an online feedback form to evaluate the effectiveness of an innovation workshop. More than 90% of participants strongly agreed or agreed that they could generate ideas to improving asthma care, the workshop helped drive innovation, and the use of photovoice helped them empathise with patients challenges. A design thinking framework can be used for innovation workshops. Photovoice is a useful method for understanding the problems faced by patients. A multidisciplinary team format with patient involvement was highly favoured.

## Introduction

Asthma is a chronic respiratory disease characterised by reversible airway obstruction. Asthma affects around 262 million people worldwide, with a prevalence of 6.6% in adults^1^. According to the Centres for Disease Control and Prevention, 61.9% of adults have poorly controlled asthma, defined as having at least one symptom more than twice a week^2^. In Singapore, the asthma prevalence is 11.9%^3^, and 87% have poorly controlled asthma^4^. Uncontrolled asthma can result in poorer quality of life^5^, higher healthcare utilisation and cost compared to well-controlled asthma^6^.

Improving asthma care in the primary care settings is important as it is where most of the care is provided^7^. Innovation workshops can be used to co-create new and creative ideas, solve problems, and improve existing processes^8^. The design thinking framework can be used to guide the development of innovations^9^. The framework was initially conceptualised by the design school to teach students to design products that meet the needs of users^10^, but has been extrapolated to the healthcare sector as it results in more successful, sustainable, and user-centred innovations while ensuring technical feasibility and economic viability^11,12^.

This paper will outline the development and execution of an asthma innovative workshop called the InnovFamLab. Design thinking framework was used to identify the problems faced by patients with asthma and to ideate creative and actionable interventions involving both healthcare providers (HCPs) and patients to improve asthma care in the primary care setting.

## Method

A one-day workshop was organised to discuss the challenges and enablers of asthma care, and ideate innovations to address the issues discussed. HCPs from SingHealth Polyclinics were purposively selected for their involvement in asthma care in the institution and their interest in innovations. The HCPs included doctors, nurses, pharmacists, clinic executives, and medical students. Invites were sent to members of the institution’s asthma workgroup, asthma champions of the clinics, and those involved in asthma-related quality improvement projects, to ensure that the HCPs were directly or indirectly involved in improving asthma care and in a position to execute changes to asthma care. Research coordinators who were interested in innovation acted as laypeople in the group. The HPCs were split into five groups, seven to eight members each, with a facilitator and a scribe. All groups comprised members of multidisciplinary HCPs to ensure that group discussions included diverse insights and perspectives^8^.

Two patients with asthma attended this workshop alongside the HCPs. The patients selected had chronic severe asthma with a history of exacerbation and were on regular follow-ups with a family physician for the last 5 years in a polyclinic. One patient had well-controlled asthma, while the other had poorly controlled asthma. This was to understand the needs of patients with varying degrees of asthma control.

This is a description of an innovation workshop using design thinking framework. These individuals are not research participants or subjects hence formal ethical approval is not required. However, we uphold the principles of informed consent, and written consent was obtained from the patients for the use of photographs in non-commercial purposes such as journal publication, training, and report writing for funders.

## Workshop framework

**Fig. 1:**
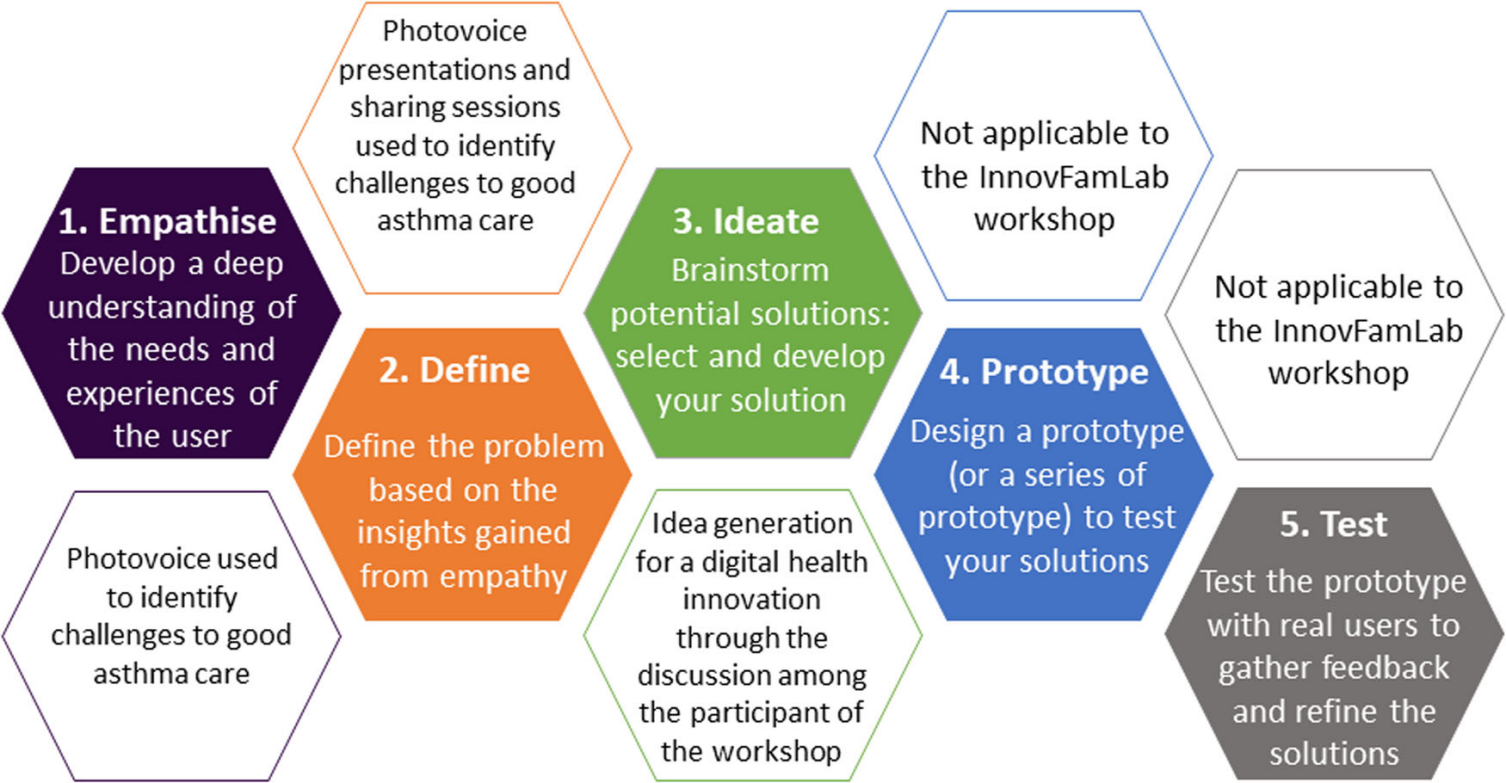
Five stages of Design Thinking Process and operationalization in the InnovFamLab workshop. Illustration of the five stages of Stanford’s d: school Design Thinking Process, definition, and operationalization in the InnovFamLab workshop.

The study team conceptualised the workshop based on the Stanford’s d: school Design Thinking Process: 1. empathise, 2. define, 3. ideate, 4. Prototype, and 5. Test^10^. It focused on the first three stages of empathise, define and ideate. Fig. 1 describes the stages of design thinking process, and how they were operationalised in this workshop.

**Fig. 2:**
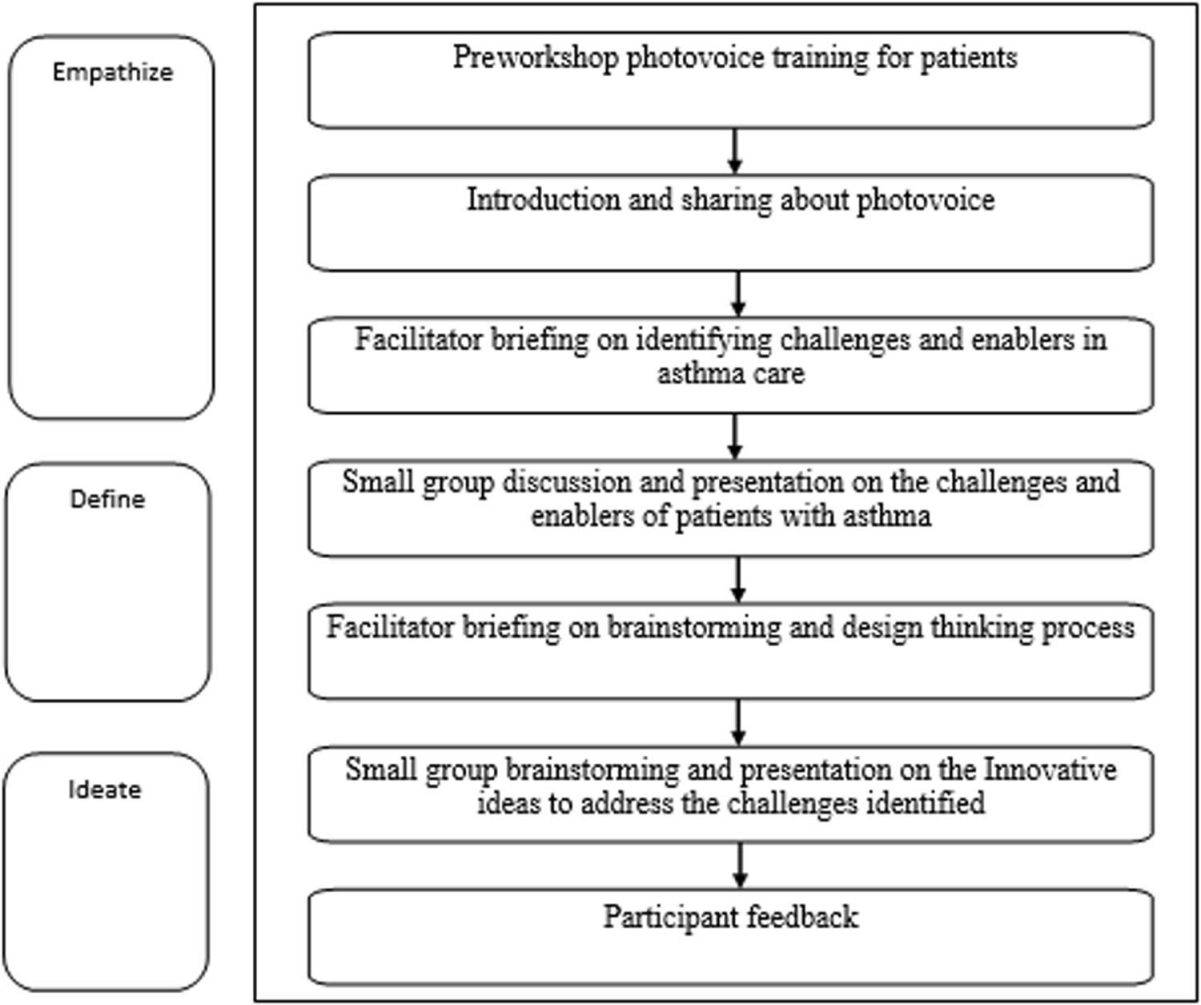
Flowchart on Workshop activities. This is the flow of the workshop activities.

The workshop flow is illustrated in Fig. 2. The empathise stage included pre-workshop training (for patients) and the introduction and sharing of photovoice to understand the challenges and enablers of patients living with asthma. The defined stage involved small group discussion and presentation on the challenges and enablers. In the ideate stage, participants brainstormed innovative ideas to address the challenges identified. A participant briefing was conducted before each small group discussion to guide the discussion.

### Pre-workshop photovoice training for patients

Photovoice is an arts-based qualitative method where people use photographs to document and share their health and reality^13^. Two patients volunteered and underwent pre-workshop photovoice training 4 weeks before the workshop by two team members. During the initial online meeting, patients were introduced to using photovoice, the ethics of photography and the tasks they needed to complete. The patients were informed to share their challenges and enablers with good asthma care using photovoice. After the initial meeting, patients were given 2 weeks to take the photographs, and the photographs were discussed during the second meeting. The third meeting was a final run-through with the patients on the photographs they were presenting at the workshop.

### Sharing by patients using photovoice

The workshop began with an introduction to photovoice. A 20-min-each presentation by the two patients using photovoice followed this, who also provided the rationale and explanation for the photographs. Only two patients were included to allow time for in-depth sharing by each participant. Afterward, the participants had a question-and-answer session to clarify the photographs.

### Briefing by facilitators

The workshop included a 10-min briefing by facilitators before each group discussion. The first briefing session guided the participants to identify the key challenges and enablers in the asthma care journey based on patients’ photovoice sharing. Participants were also advised to refrain from judging or criticising other participants’ ideas in speech or body language and encouraged to generate ‘out of the box’ ideas. The participants were introduced to the Stanford d: school Design Thinking Process during the second briefing session.

### Small group discussion

The workshop comprised two 90-min small group discussions:

1. Identifying the challenges and enablers of self-care in patients with asthma and
2. Brainstorming for innovative ideas to address the challenges identified.

A small group discussion format allowed all participants to express their thoughts and views. A facilitator was assigned to each group to guide the discussion session. One group member was allocated as a scribe to document the discussions and evaluate the group dynamics. During these group discussions, the patients went to every group to allow them to seek the patients’ opinions on their ideas.

### Group presentation

The group presentations followed the discussions. During this session, individual groups presented the outcomes of their respective small group discussion. These presentations allowed participants to share their small group discussions with the other groups and generate further discussions on the ideas. The first presentation was 10 min, and the second was 15 min.

## Data collection tools

### Group discussion and presentations

The minutes and outcomes of the small group discussions were obtained from the scribe. The findings were categorised into themes and synthesised into a report at the end of the workshop. The scribes, who were not part of the clinical team, were instructed to provide feedback on the team dynamics during discussion. This was to account for potential power distance and fear of more senior staff in the team during the team discussions^14^.

### Participants feedback form

Participants’ feedback was collected via an online feedback form to evaluate the effectiveness of this innovation workshop. Four questions were rated on a 5-point Likert scale to evaluate if they felt they had a better understanding of the challenges of patients with asthma, gained new insights to the enablers, generated ideas to improving asthma care and if the workshop helped drive innovation. Three open-ended questions obtained information on their preferences and suggestions for the workshop.

## Results

### Participant demographics

Forty HCPs were invited, and 37 attended. Three were unable to attend due to being on sick leave (*n* = 1), having personal matters (*n* = 1), and being rostered for work (*n* = 1). The demographics of the HCPs were as follows: doctors (*n* = 9, 24.3%), nurses (*n* = 14, 37.8%), pharmacists (*n* = 4, 10.8%), clinical service (*n* = 3, 8.1%), medical students (*n* = 3, 8.1%), and research staff (*n* = 4, 10.8%). Of the two patients invited, one could not present in person at the workshop as she was unwell due to asthma. Her presentation was conducted using a virtual platform, Zoom.

The study findings are summarised based on the three stages of design thinking framework: Empathise, Define and Ideate.

#### Empathise (understanding people and their challenges)

After the photovoice sharing by patients (empathise stage), 94.4% of participants strongly agreed or agreed that they had a greater understanding of the patients’ challenges in asthma care. A total of 91.6% of participants strongly agreed or agreed that they had gained new insights into the enablers of asthma patients.

#### Define (frame the problem)

From the photovoice presentation, eight challenges to good asthma care were identified. The key challenges were: 1. Executing AAP; 2. Managing triggers; 3. Participating in outdoor activities and exercise; 4. Inconveniences when travelling; and 5. Lack of public awareness of asthma triggers from social activities (e.g., smoking). The enablers were: 1. Knowing triggers; and 2. Patient’s motivations. The challenges and enablers are described in Table 1.

**Table 1:** Challenges to good asthma care.

Challenges	Description
1. Challenges in executing asthma action plan	• Language and simplicity of content. • Awareness of details in action plan. • Prompt self-identification on symptoms of exacerbation that requires stepping up of inhalers and oral steroids • Patients do not understand that they need to be on maintenance treatment and hence are less compliant. • Asthma action plans do not account for external factors and comorbidities that may concern the patient. • Lack of knowledge on asthma from not attending nurse counselling
2. Managing triggers	• Awareness and control • Triggers that patient can control • Triggers that patient cannot control
3. Outdoor activities and exercise	• Challenges to outdoor activities which has exposure to triggers • Challenges to exercise
4. Inconveniences when travelling	• Need to declare inhaler in flight and a memo from doctor to be able to carry medication in flight. Need to obtain the memo in a rush if the trip is last minute. • Not able to travel far – need to carry many medications/inhalers, limited access to healthcare, what to do if asthma trigger during travel. • Need to be aware of the changes in the weather, adjust to different weather, and be prepared for unpredictable weather.
5. Lack of public awareness of asthma triggers from social activities	• Stressful to find a good balance between maintaining good relationships with family/neighbours and maintaining good asthma control when they contribute to triggers. • Having to wear mask due to Covid-19, which causes difficulty breathing • Public awareness of asthma and management of asthma emergencies
Enablers	Description
1. Knowing triggers	• Avoiding known triggers • Family members to ensure they do not expose the patient to the triggers
2. Patient’s motivations	• Remaining symptom free • Compliance to medication

**Fig. 3:**
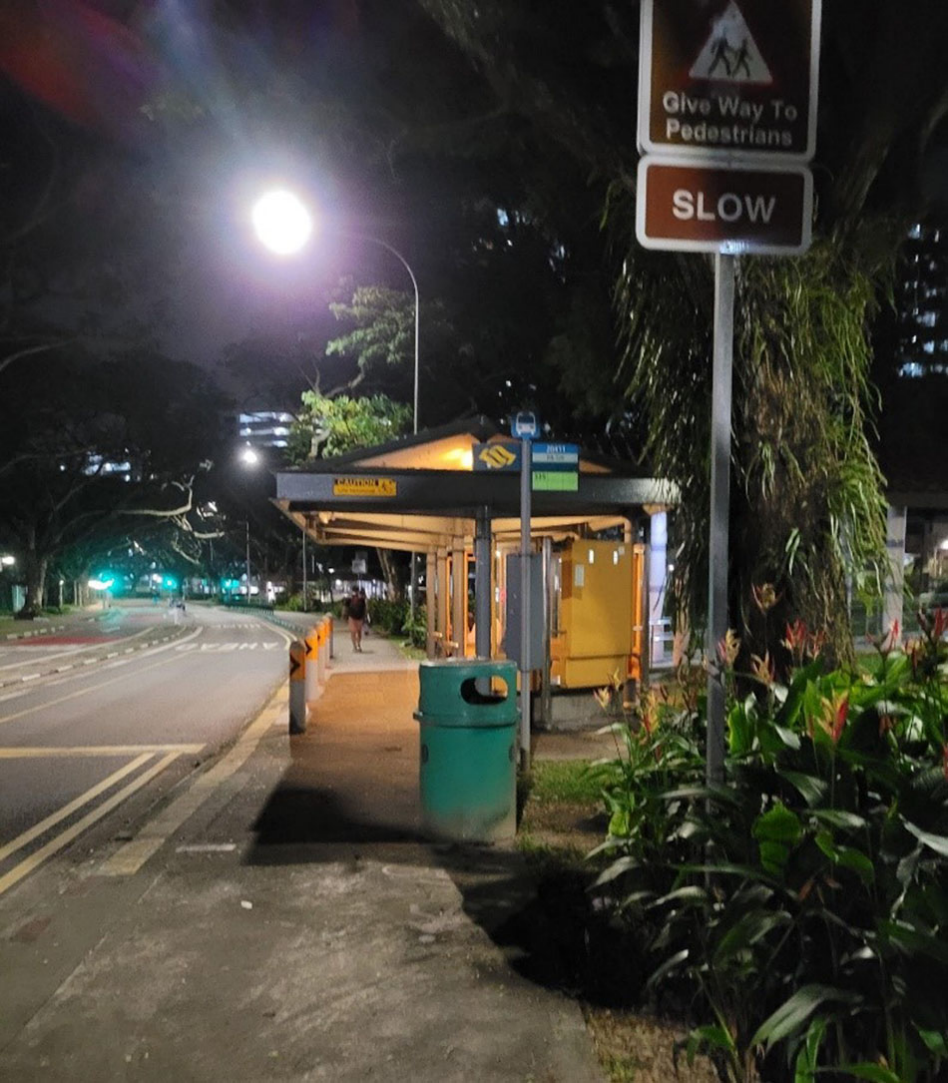
Example of a photograph. Photograph of a bus stop.

An example of using photovoice to define the problem is Fig. 3 which is a photograph of a bus stop at night. The patient used the photograph to explain that the public bus was her main mode of transport. However, there were occasionally people smoking at the bus stop while she was waiting for her bus, or left behind cigarette filters in the bin at the bus stop, which were triggers for her asthma. The patient believed that this prevalence of smoking at bus stops was due to lack of public awareness regarding the negative impact of smoking on people with asthma.

#### Ideate (Idea generation)

Six ideas to improve asthma self-care were highlighted: 1. Improve delivery of the asthma action plan (AAP); 2. Develop a symptom monitoring calendar; 3. Provide accessible travel information; 4. Develop an exercise programme for patients with asthma; 5. Promoting patient self-efficacy; and 6. Public awareness about asthma triggers and prevention.

94.4% of participants strongly agreed or agreed that they could generate ideas to improve asthma care, and the workshop helped drive innovation. Participants enjoyed listening to patients’ sharing, interactions and input, the multidisciplinary approach to facilitate group discussion, and the brainstorming session. To improve the workshop, HCPs suggested inviting more patients to share their experiences, incorporating discussions on translating the innovations to clinical setting and updating the HCP on the outcomes of the workshop discussions.

##### Feedback from scribes on team dynamics

All scribes reported that their team members actively contributed and were involved in the group discussions. The facilitators also helped by actively seeking the input of all team members.

## Discussion

This study demonstrated that incorporating the photovoice technique into the design framework of an innovation workshop helped to generate innovative ideas for improving patient care by helping workshop participants better understand the problems faced by patients. From the participants’ feedback, more than 90% of HCPs strongly agreed or agreed that they could generate ideas to improving asthma care, and the workshop helped drive innovation. In another design thinking workshop, more than 70% of participants agreed that they could apply design thinking to a problem in the clinical setting to generate potential ideas^15^.

To the best of the author’s knowledge, this is the first innovation workshop that used photovoice in the empathise step of design thinking. As ‘A picture speaks a thousand words’, photographs give context to a narrative by providing information on the environment (e.g. objects, landscape, events), relationships and norms. HCPs could visualise patients’ lives^16,17^, leading to discussion of topics that mattered to the participants^18^. From the participants’ feedback, more than 90% strongly agreed or agreed that photovoice helped them understand the challenges and enablers of good asthma care. This supported the use of photovoice in the empathise step of design thinking.

Stakeholder engagement allows interventions to cater to the preferences of both patients and care providers, which are then more likely to improve patient care and outcomes^19^. The stakeholders’ feedback that they favoured the multidisciplinary team approach to group allocation. Being in different professions, they often did not know the work processes of the other departments. As the operation and logistical challenges could pose a barrier to the interventions, it enabled them to understand the challenges faced by the different departments in executing the intervention. Such team grouping opened discussions on tackling these challenges from a clinical and logistics perspective.

The HCPs appreciated having patients present and share their perspectives during the workshop and suggested inviting more patients to such events in the future. Increasing involvement of laypeople in healthcare research can provide information on users’ expectations and experiences^20^. This study is novel as involvement of patients was less common in developing technological innovations^21^. It is also vital that stakeholder engagement remains a continuous activity to seek feedback on the interventions developed and to keep them informed on the discussion outcomes. A limitation with inviting patients to the discussion could be related to confidentiality issues if the patient did not want to be identified and the inability to anonymise if sensitive topics were raised.

The strength of this workshop was the participation of multidisciplinary teams and patient advocates to co-create innovative ideas that have meaningful outcomes for patients. As this was a 1-day workshop, the prototype and test stage of the design thinking framework was not achieved. We plan to adopt the Medical Research Council framework for evaluating complex interventions^22,23^. This framework emphasises the importance of a phased approach, including the development, feasibility, and evaluation stages. By incorporating the prototype and test stages into our future work, we aim to gather more comprehensive data on the effectiveness and practicality of the proposed solutions. This iterative process will allow us to refine and optimise the interventions based on real-world feedback and ensure their relevance and applicability in practice.

While the design thinking framework is widely used, the integration of the Photovoice method brings a novel and valuable dimension to intervention development. Its participatory nature, generation of rich qualitative data, user-centred approach, and collaborative decision-making contribute to the depth, relevance, and authenticity of the interventions. We believe that the inclusion of Photovoice enhances the workshop’s uniqueness and strengthens the overall quality of the intervention development process.

We acknowledge the limitations of having only two patients involved in the workshop, which limited the breadth of patients’ experiences shared. Despite this, the photographs allowed for a deep exploration of the topic, with rich and detailed insights obtained from patients’ perspectives and experiences expressed through photographs. In addition, the workshop team spent a significant time with each patient to foster trust and rapport. This facilitated the collection of detailed and nuanced findings, uncovering the complexities and intricacies of the patients’ perspectives.

One of the challenges of a face-to-face workshop is the patient not being able to turn up last minute. Thus, it is essential to have contingency plans. An alternative would be to record the patient’s presentation before the workshop as a backup.

A design thinking framework can be used for innovation workshops. Photovoice is a useful method for understanding the problems faced by patients. A multidisciplinary team format with patient involvement was highly favoured. This framework could be considered in the design of future innovation workshops for other health conditions and settings.

### Reporting Summary

Further information on research design is available in the Nature Portfolio Reporting Summary linked to this article.

### Supplementary information

reporting summary checklist
